# Sleep Problems and New Occurrence of Chronic Conditions during the COVID-19 Pandemic in the UK

**DOI:** 10.3390/ijerph192315664

**Published:** 2022-11-25

**Authors:** Jane Falkingham, Maria Evandrou, Athina Vlachantoni, Min Qin

**Affiliations:** 1 ESRC Centre for Population Change (Connecting Generations), Faculty of Social Sciences, University of Southampton, Southampton SO17 1BJ, UK; 2 Centre for Research on Ageing, Faculty of Social Sciences, University of Southampton, Southampton SO17 1BJ, UK

**Keywords:** sleep, physical health, mental health, COVID-19 pandemic

## Abstract

The COVID-19 pandemic has negatively impacted upon sleep health. Relatively little is known about how this may influence the population’s health subsequently. This prospective longitudinal study aims to examine the consequences of sleep problems for physical and mental health during the COVID-19 pandemic in the UK, using data from the Understanding Society: COVID-19 Study, a large-scale population-based survey with 12,804 adults aged 16 and above. A measure of sleep problems was derived from Pittsburgh Sleep Quality Index (PSQI) questions, reflecting seven dimensions of sleep quality. Binary logistic regressions were applied to investigate the relationship between sleep problem and the incidence of cardiovascular disease (CVD), hypertension, diabetes, obesity, arthritis and an emotional, nervous or psychiatric problem over the 15 months follow-up period. The analysis confirms that sleep problems are prevalent and vary between sub-groups among adults. Notably, sleep problems are then significantly associated with a higher risk of cardiovascular disease, hypertension, diabetes, obesity, arthritis and emotional, nervous or psychiatric problems, independent of demographic, socioeconomic, familial and health confounders. Our findings suggest promoting good sleep hygiene should be prioritised during the pandemic, and more generally as part of wider programmes aimed at promoting good physical and mental health.

## 1. Introduction

Globally, the COVID-19 pandemic is having a notable impact on sleep health due to the psychological stressors brought on by the coronavirus itself and the social isolation measures implemented to manage the virus. Sleep problems have been found common during the pandemic and have been associated with higher levels of psychological distress [1]. The UK entered a state of lockdown on 23 March 2020, in an unprecedented effort to stop the spread of the coronavirus, with the government mandating all those who could to work from home, closing schools, restaurants and all but essential shops, and advising the population to stay at home and limit contact with other individuals outside their household. It is reported that over half of the population struggled to sleep during this first lockdown [2] and elevated sleep loss since the pandemic has been observed to be higher compared to levels pre-pandemic [3]. Moreover, the pandemic has widened sleep deprivation disparities, with women with young children, those who have experienced COVID-19 infection and Black, Asian and individuals from minority ethnic (BAME) communities all experiencing a heighten risk of sleep loss, which may adversely affect their mental and physical health [3].

The 3P behavioral model of insomnia [4], which describes how acute insomnia develops and how acute insomnia becomes chronic and self-perpetuating, has gained considerable recognition. The interaction of three factors is the model’s foundation. The predisposing and precipitating factors, such as worry, child-rearing, and life-stressing occurrences, including physical and mental illness, provide a stress-diathesis perspective of how insomnia manifests. The perpetuating factor, such as the behaviors an insomniac person adopts in order to cope with their lack of sleep, shows how behavioral factors modify chronicity.

Sleep is essential for maintaining healthy systemic physiology across the body systems, including the brain. Sleep problems or dysfunction have significant long-term effects on psychological and physical health [5], with potential biological mechanisms being associated with increased activity of the sympathetic nervous system and hypothalamic–pituitary–adrenal axis, metabolic effects, changes in circadian rhythms, and pro-inflammatory responses [6]. Previous empirical studies have found that sleep disruptions have substantial adverse health consequences and increase the risk of cardiovascular diseases [7], hypertension [8], type 2 diabetes [9], obesity [10], arthritis [11] and depression [12].

The majority of previous studies have been conducted in clinical settings, with relatively little data on the relationship between sleep problems and chronic conditions in community settings. Furthermore, there is to date limited evidence on how sleep problems experienced during the COVID-19 pandemic may subsequently impact the population’s health. This study aims to fill this research gap by exploring a community-based longitudinal study to examine the consequences of sleep problems on physical and mental health during the COVID-19 pandemic in the UK.

## 2. Materials and Methods

### 2.1. Study Design and Setting

This study adopted a prospective longitudinal design, analysing data from the Understanding Society (USoc): COVID-19 Study, a large-scale population-based study [13]. The observation period was from July 2020 to September 2021, covering 15 months during the COVID-19 pandemic in the UK. The key independent variable, i.e., experiencing sleep problems, and all demographic and socio-economic covariates were measured in July 2020. The outcomes, which were the new occurrence of selected chronic conditions, were measured over the subsequent 15 months. Details of all the variables are provided in the measures section below. 

Understanding Society (USoc) is an ongoing panel survey of more than 40,000 households, commencing with the initial round of data collection in 2009. Between 24 and 30 April 2020, members of households who had participated in either of the two most recent main USoc data collections (rounds 8 and/or 9) and who were older than 16 years were invited to complete the first wave of the COVID-19 survey totalling 42,330 sample members. Data were collected online in nine subsequent waves—wave 1 (April), wave 2 (May), wave 3 (June), wave 4 (July), wave 5 (September), wave 6 (November) 2020, wave 7 (January), wave 8 (March) and wave 9 (September) 2021. The probability sample was drawn from postal addresses. Northern Ireland and areas in England, Scotland and Wales with proportionately large migrant and ethnic minority populations were oversampled. In waves 2 to 4, everyone eligible in wave 1 continued to be eligible irrespective of whether they had participated in any of the previous surveys. From the September 2020 (wave 5) survey onwards, only sample members who had completed at least one partial interview in any of the first four web surveys were invited to participate. From the November 2020(wave 6) survey onwards, the survey team stopped inviting those who had only completed the initial survey in April 2020 and none since. The response rate (full interview) for each of the nine waves of the USoc: COVID-19 Study was 39%, 35%, 33%, 32%, 30%, 28%, 28%, 30% and 30%, respectively [14] (Table 6.3, page 14).

### 2.2. Participants

This study included all participants aged 16 and above who participated USoc: COVID-19 Study in July (wave 4) and any subsequent surveys (wave 5, wave 6, wave 7, wave 8 and wave 9). Wave 4 (July 2020) is used as the baseline for this study as the wave 4 survey questionnaire included a detailed set of sleep-related questions. The timing of this round of data collection in July 2020 was scheduled just after the restrictions of the UK’s first lockdown were being lifted.

**Figure A1:**
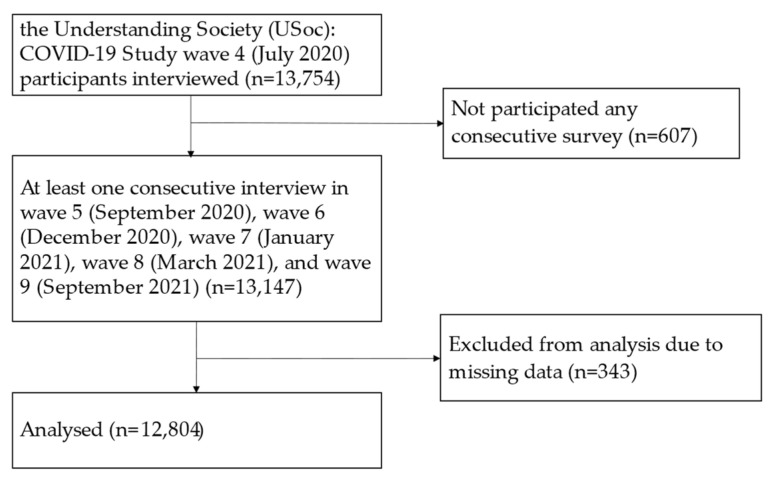
The enrolment of respondents and the selection procedure.

In wave 4, 13,754 respondents participated in the survey. During the consecutive wave 5, wave 6, wave 7, wave 8 and wave 9, 13,147 participants attended at least one more survey; 607 did not respond to any further wave and as such were excluded from the study. Further, 342 participants were excluded from the analysis due to missing data in the dependent variables or the study chronic disease data. Missing values of covariates were imputed using chained equations [15]. The final analytical sample comprised 12,804 respondents. It is notable that the majority of respondents participated in both wave 4 and wave 9 (12,574 out of 12,804) and had 15 months of observations. The overall mean age was 48.9 years (SD: 18.6; range: 16–98), and 52.9% were female. A flowchart showing the enrolment of respondents and the analytical sample selection procedure is presented in Appendix A Figure A1.

### 2.3. Measures

#### 2.3.1. Sleep Problems

The key independent variable of sleep problems was derived from a short Pittsburgh Sleep Quality Index (PSQI) questionnaire. In July 2020, all USoc: COVID-19 Study participants were asked seven questions (listed at the end of this section) about their usual sleep habits during the past month. These questions were drawn from the original PSQI questionnaire [16], reflecting seven dimensions of sleep quality—subjective sleep quality, sleep latency, sleep duration, habitual sleep efficiency, sleep disturbances, use of sleeping medication and daytime dysfunction. Based on the participants’ answers to each question, a score ranging from 0–3 points was awarded (described following each question and options at the end of this section). In all cases, a score of 0 indicated no problem, while a score of 3 indicated a severe problem. The internal consistency for the seven dimensions’ sleep quality scores was acceptable, with Cronbach’s alpha equal to 0.74. The value of alpha should be above 0.70 [17]. The seven component scores were then added to yield one global score, ranging from 0 to 21 points. A score of more than 5 suggests poor sleep quality, and a score of equal or less than 5 suggests good sleep quality. 

The psychometric properties of the original PSQI consist of 19 self-rated questions and five questions rated by the ‘bed partner’ or roommate are well documented, and the index can be used as a screening measure to identify sleep dysfunction [18]. Moreover, published findings have outlined high correlations of the PSQI with other measures of sleep quality, including clinical diagnosis of insomnia [19]. The length of the PSQI may limit its utility and application for some types of studies. Certain short versions, e.g., 13-item or 6-item forms, have been previously developed (validated and psychometrically tested) with the purpose of reducing the burden of extensive surveys [20,21].

Based on the severity of the sleep disruptions and referring to the classification used in a previous study [22], respondents were then grouped into three subgroups: good sleeper, moderate sleep problems, and severe sleep problems. Severe sleep problems were characterised by reporting very bad sleep quality (Q7) (score of 3 on a scale of 0–3) with their sleep patterns and (i) reporting symptoms of the initial (Q2) or (ii) middle, or late insomnia (Q3) at least 3 nights per week for the last month or (iii) reporting substantial daytime impairment related to sleep difficulties (Q6) (score of 3 on a scale of 0–3). A previous study subgrouping poor sleep quality found the individuals using sleep medication had the lowest Pittsburgh Sleep Quality Index total scores but had similar mental and physical comorbid patterns as the high insomnia group [23]. Therefore, participants were also automatically classified into the group with severe sleep problems if they used prescribed or over-the-counter sleep medicine at least 3 nights per week (Q5), regardless of their answers to the other questions. 

Those classified as having moderate sleep problems had a global score greater than 5 but did not meet the above criteria for severe sleep problems. The rest of the participants, with a global score equal to or less than 5, were classified as “good sleepers”. 

Sensitivity analysis was conducted using an alternative conventional classification based on using the threshold of PSQI global score greater than 5 to distinguish between good sleep and poor sleep quality for all respondents [18]. These results were then compared with the main analyses to explore any dose-effects of sleep problems on the health consequences.

The following questions relate to your usual sleep habits during the last month:

Q1. How many hours of actual sleep did you usually get per night during the last month? This may be different than the number of hours you spent in bed. 

Score 0 (>7 h), Score 1 (6–7 h), Score 2 (5–6 h), Score 3 (<5 h)

Q2. During the past month, how often have you had trouble sleeping because you cannot get to sleep within 30 min?

1. Not during the past month
2. Less than once a week
3. Once or twice a week
4. Three or more times a week
5. More than once most nights

Score 0 (option 1), 1 (option 2), Score 2 (option 3), Score 3 (option 4 or 5)

Q3. During the past month, how often have you had trouble sleeping because you wake up in the middle of the night or early in the morning?

1. Not during the past month
2. Less than once a week
3. Once or twice a week
4. Three or more times a week
5. More than once most nights

Score 0 (option 1), Score 1 (option 2), Score 2 (option 3), Score 3 (option 4 or 5)

Q4. During the past month, how often have you had trouble sleeping because you cough or snore loudly?

1. Not during the past month
2. Less than once a week
3. Once or twice a week
4. Three or more times a week
5. More than once most nights

Score 0 (option 1), Score 1 (option 2), Score 2 (option 3), Score 3 (option 4 or 5)

Q5. During the past month, how often have you taken medicine (prescribed or “over the counter”) to help you sleep?

1. Not during the past month
2. Less than once a week
3. Once or twice a week
4. Three or more times a week

Score 0 (option 1), Score 1 (option 2), Score 2 (option 3), Score 3 (option 4)

Q6. During the past month, how often have you had trouble staying awake while driving, eating meals, or engaging in social activity?

1. Not during the past month
2. Less than once a week
3. Once or twice a week
4. Three or more times a week

Score 0 (option 1), Score 1 (option 2), Score 2 (option 3), Score 3 (option 4)

Q7. During the past month, how would you rate your sleep quality overall?

1. Very good
2. Fairly good
3. Fairly bad
4. Very bad

Score 0 (option 1), Score 1 (option 2), Score 2 (option 3), Score 3 (option 4)

Source: University of Essex, Institute for Social and Economic Research, 2021

#### 2.3.2. Disease Incidence

In each wave of the survey, participants were asked whether they had been diagnosed with chronic disease using the question: “Has a doctor or other health professional ever told you that you have any of the conditions listed on this card?” The disease list included coronary heart disease, heart attack or myocardial infarction, angina, stroke, hypertension, diabetes, obesity (having a Body Mass Index (BMI) of 40 or above), arthritis, an emotional, nervous or psychiatric problem, amongst others. 

The accumulative self-report of diagnosed chronic diseases at wave 4 was considered as the baseline. Any new occurrence of the listed conditions reported in the subsequent surveys was then taken to represent a new incidence of physical or mental illness. For each chronic condition, participants reporting a new diagnosis of the condition at wave 5, wave 6, wave 7, wave 8 or wave 9 coded as 1, and those who did not report a new diagnosis of the condition were coded as 0. The new medical condition was measured by the presence of a diagnosed chronic condition at the survey time, given that it was absent at the baseline (July 2020). Because chronic conditions may not be curable over a short time, the longer the observation period, the more newly diagnosed chronic disease cases were accumulated. Take the new occurrence of Hypertension as an illustration. By waves 5, 6, 7, 8, 9, the number of accumulated new occurrences was 37, 80, 118, 140 and 176, respectively. If later waves were missed out, e.g., 8–9, we would expect a slight underestimation of a new occurrence of the chronic condition. 

For the purposes of this study, diagnoses of coronary heart disease, myocardial infarction, angina and stroke were grouped together under cardiovascular disease (CVD). Reporting of a new incidence of six diseases were then used as the outcome variables for the analyses. These were CVD, hypertension, diabetes, obesity, arthritis and an emotional, nervous or psychiatric problem. 

#### 2.3.3. Control Variables

Drawing upon the extensive literature relating to the social determinants of chronic disease [24,25], the analyses included a number of control variables relating to the demographic (age, gender, ethnicity, living with a partner, dependent children in the household) and socioeconomic (educational qualification, National Statistic Socio-economic classification (NS-SEC), perceived current and future financial difficulties) characteristics of the respondents. In addition, the analysis also controlled for reporting of suffering from COVID-19 symptoms and a number of other chronic conditions at baseline (Emphysema, Chronic bronchitis, Chronic Obstructive Pulmonary Disease, Cystic fibrosis, Hypothyroidism or an under-active thyroid, Any kind of liver condition, Cancer or malignancy, Epilepsy, Multiple Sclerosis, H.I.V., Chronic kidney disease, Conditions affecting the brain and nerves, such as Parkinson’s disease, motor neurone disease, a learning disability or cerebral palsy, Problems with your spleen or you’ve had your spleen removed, Sickle cell disease, Other long standing/chronic condition). 

### 2.4. Statistical Analysis

Binary logistic regression was used to investigate the relationships between sleep problems at baseline and the incidence of a number of selected diseases, i.e., CVD, hypertension, diabetes, obesity, arthritis and an emotional, nervous or psychiatric problem. Initial analyses of men and women separately led to a very low number of new incidents of some diseases, so the decision was taken to run the models with men and women combined and including gender as a control variable. Participants who had previously been diagnosed with the disease of interest at wave 4 were excluded from the specific multivariate analysis.

Three sets of models were run for each of the six diseases. Model 1 included just the variable pertaining to sleep problems as the predictor; Model 2 then adjusted for age, gender and ethnicity; Model 3 further adjusted for education, NS-SEC, perceived current financial difficulty, perceived future financial difficulty, living with a partner, a dependent child under 16 in the household, COVID-19 symptoms, and any other baseline diagnosed chronic diseases.

Two sets of sensitivity analyses were conducted to check the robustness of the results. First, using the core measure of sleep problems, respondents who did not participate in wave 9 were excluded; in this instance, the analytical sample was restricted to respondents who participated in both wave 4 and wave 9 surveys and thus had a full 15 months of observation time. This yielded an analytical sample of 12574 participants. The second set of sensitivity analyses then used the full analytical sample but included an alternative dichotomous variable of sleep problems based on the threshold of PSQI global score greater than 5 to distinguish all participants between good sleep and poor sleep quality [18].

## 3. Results

### 3.1. Sample Characteristics and Bivariate Analysis Results

Table 1 shows the characteristics of the sample and bivariate association with the level of sleep problems. Among the UK population surveyed in July 2020, over half were good sleepers, more than one-third (39.1%) had moderate sleep problems, and less than one in ten (8.1%) had severe sleep problems.

Sleep problems were not evenly distributed in the population but were significantly associated with a range of demographic, socioeconomic, and familial characteristics and health conditions during the pandemic. Women reported a higher level of moderate and severe sleep problems than men. Sleep problems were also higher amongst those in mid-life (aged 45–64) in comparison to other age groups. Individuals from Black, Asian and other minority ethnic communities and Non-British White also recorded a higher prevalence of severe sleep problems compared to those reporting themselves as ‘British White’. Individuals with lower socioeconomic status and financial difficulties also recorded a higher proportion of moderate and severe sleep problems. Participants living with a dependent child had a higher proportion of moderate sleep problems, whilst those who did not live with a partner had more severe sleep problems. Finally, participants with COVID-19 symptoms or baseline chronic disease had a higher proportion of moderate and severe sleep problems. Among participants with COVID-19 symptoms, 49.3% reported moderate sleep problems, and 13.6% reported severe sleep problems. The figures are higher than the corresponding figures among those without COVID-19 symptoms (38.9% and 7.9%, respectively). The difference is statistically significant (*p* < 0.001). It is important to note that this table reports bi-variate relationships, and it is recognised that the prevalence of severe sleep problems may be higher where some of these characteristics intersect. 

Over the 15 months observation period during the pandemic, 78 (0.7%) participants reported being newly diagnosed with a CVD, 176 (1.7%) with hypertension, 66 (0.6%) with diabetes, 121 (1.0%) with obesity, 216 (1.9%) with arthritis, and 129 (1.1%) were diagnosed with an emotional, nervous or psychiatric problem. Individuals’ level of sleep problems, demographic, socioeconomic, familial characteristics and health conditions are associated with the chance of newly occurring chronic diseases (Appendix A Table A1). 

### 3.2. Logistic Regressions Results

Table 2 shows the odds ratios for disease incidence for those respondents classified as having moderate or severe sleep problems compared with good sleepers. The total number of participants in the analyses varies across the different chronic diseases because the number of excluded participants who reported being diagnosed with a particular condition at baseline differs across six diseases. After adjusting for age, sex and ethnicity (Model 2), there were significant associations between moderate and severe sleep problems and the risk of CVD, hypertension, obesity, arthritis and an emotional, nervous or psychiatric problem. The risk of developing diabetes was also significantly associated with severe sleep problems but not moderate sleep problems.

The associations between severe sleep problems and risk of CVD, hypertension, diabetes, obesity, arthritis and an emotional, nervous or psychiatric problem remained significant after additional adjustment for education, NS-SEC, perceived current financial difficulty, perceived future financial difficulty, living with a partner, a dependent child under 16 in the household, COVID-19 symptoms, and any other baseline diagnosed chronic diseases (Model 3 in Table 2). In the fully adjusted model, the association between moderate sleep problems and risk of hypertension, obesity, arthritis and an emotional, nervous or psychiatric problem remained significant, but the risk of CVD was not significant (Model 3 in Table 2).

Sensitivity analyses conducted on a restricted analytical sample, limited only to participants with 15 months of observations, showed similar results to the main analysis (Appendix A Table A2). Additional sensitivity analyses using an alternative dichotomous variable of sleep problems (Appendix A Table A3) resulted in odds ratios that were greater in magnitude to those found for moderate sleep problems in the main analysis but smaller than those severe sleep problems. The association between poor sleep quality and incidence of diabetes in this additional analysis was not significant after adjusting for socioeconomic, familial and health confounders, suggesting that the three-category classification of sleep problems provides a better gradient in predicting the risk of chronic diseases than the two-category classification.

## 4. Discussion

Our results indicate that sleep problems are prevalent and vary between sub-groups among adults in the UK during the pandemic. Moreover, sleep problems are related to an increased risk of a broad range of cardiovascular, metabolic and mental chronic conditions. These associations were observed independent of demographic, socioeconomic, familial and health confounders.

Sleep problems are common among the general population, with numerous contributing factors, from lifestyle and environmental factors to psychosocial issues [26]. Prior to the pandemic, research found that people may have sleep issues when they encounter major public health threats [27,28], findings that were then confirmed during the first months of the COVID-19 pandemic. The high prevalence and non-even distribution of sleep problems in July 2020 found in this study, with more severe problems being reported by people from a lower socioeconomic position, women, those with young children, those infected with COVID-19 and BAME individuals, are consistent with other research [3,29,30]. Possible explanations include fear of COVID-19 and sleep-related factors, such as altered sleep-wake patterns with delayed bedtime and sleep start time owing to quarantine and lockdown [31]. Difficult living conditions induced chronic stress, racism, risky behaviours and poor health status have also been proposed as potential factors underlying the pathway between socioeconomic position and inequalities in sleep health [29,30]. 

Sleep is essential for critical physiological functions to support good health. Through the biological linkage to pro-inflammatory reactions, metabolic impacts, increased sympathetic nervous system activity, abnormalities in circadian rhythms, and the hypothalamic-pituitary-adrenal axis, sleep problems have a high potential to negatively impact both short- and long-term health in both otherwise healthy people and people with underlying medical conditions [6]. It should be noted that it may take years to develop a chronic disease. Predisposing, precipitating, and perpetuating factors may in turn influence the development of other health outcomes, such as sleep problems, through physiological and psychological mechanisms during the life course from childhood until later life. Chronic conditions such as CVD, metabolic diseases and mood disorders may be associated with this progressive deterioration in people’s global health, and sleep problems can be a good indicator of such decline. This study confirms that sleep problems can present independently or alongside other medical conditions or mental health disorders and that it is a risk factor for developing these other disorders if not treated [32]. 

Most previous studies of sleep problems and their health consequences have focused on individual diseases such as hypertension, type 2 diabetes or depression [8,9,12]. This study has explored the association between sleep problems and the risk of 6 types of chronic conditions in a single dataset providing information about the broad impact of specific exposures. Moreover, this community-based longitudinal study was conducted during the unique context of the COVID-19 pandemic and covered all adults aged 16 and above in the UK. We used an index capturing a comprehensive measure of a range of sleep problems, including sleep duration, quality, timing, and regularity.

## 5. Limitations

A number of limitations within the study need to be taken into consideration. First, the follow-up period was limited to 15 months. The impact of the COVID-19 pandemic on sleep health among different sub-groups is likely to have a longer-term adverse impact on physical and mental health and it is important that future studies continue to monitor such consequences. 

Second, the diagnosed physical and mental chronic conditions were self-reported, and there is the possibility that recall bias may introduce measurement errors. Linked individual administrative health records, such as GP and hospital records, could help to avoid such bias. Moreover, health-seeking behaviour has altered during the pandemic. It is reported that after the pandemic began, primary care consultations significantly decreased in England, and a range of chronic conditions saw a sharp decline in terms of diagnoses. Individuals in the most socioeconomically disadvantaged groups have experienced greater adverse health impacts [33]. As a result, participants with sleep problems may be less likely to seek a doctor’s help and have new diseases diagnosed and so our study underestimates the impact on chronic health. It is also possible however that such newly diagnosed conditions (even if not discovered yet) may have caused sleeping problems in the first place. Future studies should consider these issues. 

Lastly, the study focusses on the impact of sleep problems reported in July 2020 over the subsequent 15 months. There is no information on how long the sleep problems lasted and whether the problems were situational, recurrent, or chronic. According to the natural history of insomnia, some episodes of sleep problems will persist, and some will remit over time. Some previous studies have found that sleep problems such as insomnia are often persistent, particularly when it reaches the diagnostic threshold for an insomnia disorder [22]. Going forward, one recommendation is that future rounds of the main Understanding Society survey and other sleep health related surveys include questions around duration in addition to the PSQI core question, which relate to experiences in the last month. Given the 15 months of observations, we acknowledge the small number of incidences of each condition that might reduce the power of the analysis. However, the association between sleep problems and incidences of chronic conditions are strong.

## 6. Conclusions

In conclusion, the COVID-19 pandemic was substantially associated with the presence of sleep problems in the UK during its first phase (July 2020). Those in a lower socioeconomic position, facing financial difficulties, women, BAME individuals, and those with baseline health conditions—including COVID-19 infections, were all more likely to report sleep problems. The novel, and alarming, finding of this study is that experiencing moderate or severe problems with sleep in July 2020 is associated with a higher risk *over the subsequent 15 months* of being diagnosed with cardiovascular disease, hypertension, diabetes, obesity, arthritis and an emotional, nervous or psychiatric problem—even after controlling for demographic, socioeconomic, familial and other health factors. This suggests that the shadow of the disruption to individual lives experienced during the first phase of COVID-19 pandemic will continue to be felt over the medium to long term.

Promoting good sleep hygiene needs to be prioritised during the continuing COVID-19 pandemic and should be included in the planning for any future public health emergency, targeting people at high risk. Cognitive behavioral therapy for insomnia has a strong track record of helping people with sleep issues and changes in sleep patterns durable over time [4]. The treatment is based on the 3P behavioral model of insomnia—predisposing, precipitating, and perpetuating factors that all contribute to developing and maintaining chronic insomnia. Given the clear association between poor sleep health and other health outcomes, basic information that can help with sleep issues, such as altering sleeping habits and regular daytime exercise should be made available in public places in the same way as other key public health messages promoting good health such as hand washing. Otherwise, we run the risk of a hidden ‘sleep health pandemic’ resulting in adverse physical and mental health outcomes continuing well beyond the initial impact of COVIID-19 has retreated.

## Figures and Tables

**Table 1:** Characteristics of the sample and bivariate association between characteristics and the level of sleep problems.

	Row % of Sleep Problems			*p* Value	% Among All Respondents
	Good Sleeper	Moderate Sleep Problems	Severe Sleep Problems		
Total	52.8 (6948)	39.1 (4950)	8.1 (906)		100.0 (12,804)
Mean age	48.5	49.2	50.1	0.022	48.9 years
Age group				<0.001	
16–24	60.2	35.0	4.7		12.7
25–44	51.1	40.5	8.4		28.4
45–64	50.4	40.1	9.5		35.9
65–79	54.3	38.7	7.0		18.9
80+	55.7	36.3	8.0		4.1
Gender				<0.001	
Men	59.2	34.8	6.0		47.0
Women	47.1	43.0	9.9		52.9
Ethnicity				<0.001	
British white	52.4	39.8	7.8		86.3
Other white	52.9	37.2	9.9		3.4
BAME	54.4	35.6	10.0		7.8
Education				<0.001	
No qualification	39.2	44.5	16.3		6.7
GCSE	50.4	41.5	8.1		28.4
A level	53.0	38.6	8.3		21.0
Degree or above	56.2	37.1	6.7		38.6
NS-SEC				<0.001	
Professional	56.4	38.7	5.0		23.3
Intermediate	56.0	38.5	5.5		12.1
Routine	50.7	41.4	7.9		18.5
Not working	50.2	38.9	10.9		40.7
Perceived current financial difficulty				<0.001	
Living comfortably	63.4	31.7	4.9		26.5
Doing all right	55.5	39.5	5.0		46.3
Just getting by	39.3	47.3	13.4		19.1
Difficulty or very difficult	15.7	54.5	29.8		6.2
Perceived future financial difficulty				<0.001	
No change	53.7	38.9	7.4		79.1
Better off	55.3	39.7	5.0		9.0
Getting worse	36.1	47.1	16.8		10.0
Living with a partner				<0.001	
No	50.2	38.1	11.7		40.2
Yes	54.5	39.9	5.6		59.8
Dependent child under 16 in the household				<0.001	
No	53.3	38.2	8.5		78.1
Yes	50.9	42.6	6.5		21.9
COVID-19 symptoms				<0.001	
No	53.2	38.9	7.9		97.8
Yes	37.1	49.3	13.6		2.2
Baseline chronic disease					
CVD	48.7	35.9	15.4	<0.001	5.6
Without CVD	53.1	39.3	7.6		94.4
Hypertension	46.9	42.2	10.9	<0.001	16.0
Without Hypertension	53.9	38.6	7.5		84.0
Diabetes	43.2	39.8	17.0	<0.001	6.9
Without Diabetes	53.5	39.1	7.4		93.1
Obesity	28.7	51.4	19.9	<0.001	4.5
Without Obesity	53.9	38.6	7.5		95.5
Arthritis	38.4	45.1	16.4	<0.001	12.0
Without Arthritis	54.8	38.3	6.9		88.0
An emotional, nervous or psychiatric problem	33.2	41.8	25.0	<0.001	6.2
Without An emotional, nervous or psychiatric problem	54.1	39.0	6.9		93.8

Source: USoc: COVID-19 Study wave 4, Weighted % and non-weighted *n*. *p*-value based on ANOVA test to compare the mean age of different sleep problems and Pearson chi-square tests for the association between all other characteristic variables and sleep problems.

**Table 2:** Odds ratios (95% Confidence Intervals and *p* Values) for incidents of chronic disease according to the level of sleep problems over 15 months during the COVID-19 pandemic.

Incidents of Chronic Disease	Total *n*	Sleep Problems		
		Good Sleeper	Moderate Sleep Problems	Severe Sleep Problems
CVD (*n* = 78)	12,123			
Model 1		1.00	1.41 (0.87–2.27) (*p* = 0.159)	2.34 (1.15–4.75) (*p* = 0.019)
Model 2		1.00	1.68 (1.04–2.73) (*p* = 0.035)	3.01 (1.46–6.20) (*p* = 0.003)
Model 3		1.00	1.54 (0.95–2.51) (*p* = 0.083)	2.64 (1.25–5.54) (*p* = 0.011)
Hypertension (*n* = 176)	10,420			
Model 1		1.00	1.88 (1.37–2.60) (*p* < 0.001)	2.81 (1.73–4.58) (*p* < 0.001)
Model 2		1.00	1.99 (1.43–2.75) (*p* < 0.001)	3.01 (1.83–4.95) (*p* < 0.001)
Model 3		1.00	1.88 (1.35–2.61) (*p* < 0.001)	2.54 (1.51–4.26) (*p* < 0.001)
Diabetes (*n* = 66)	11,895			
Model 1		1.00	1.54 (0.90–2.63) (*p* = 0.115)	3.86 (1.94–7.67) (*p* < 0.001)
Model 2		1.00	1.58 (0.92–2.71) (*p* = 0.096)	3.94 (1.96–7.91) (*p* < 0.001)
Model 3		1.00	1.41 (0.82–2.44) (*p* = 0.219)	2.72 (1.29–5.72) (*p* = 0.009)
Obesity (*n* = 121)	12,229			
Model 1		1.00	1.83 (1.24–2.70) (*p* = 0.002)	3.19 (1.82–5.59) (*p* < 0.001)
Model 2		1.00	1.73 (1.17–2.56) (*p* = 0.006)	2.92 (1.66–5.14) (*p* < 0.001)
Model 3		1.00	1.58 (1.06–2.35) (*p* = 0.023)	2.13 (1.08–3.61) (*p* = 0.014)
Arthritis (*n* = 216)	11,085			
Model 1		1.00	1.73 (1.30–2.30) (*p* < 0.001)	2.76 (1.76–4.33) (*p* < 0.001)
Model 2		1.00	1.79 (1.34–2.40) (*p* < 0.001)	3.07 (1.93–4.87) (*p* < 0.001)
Model 3		1.00	1.65 (1.23–2.21) (*p* = 0.001)	2.42 (1.50–3.91) (*p* < 0.001)
An emotional, nervous or psychiatric problem (*n* = 129)	12,052			
Model 1		1.00	1.98 (1.33–2.95) (*p* < 0.001)	6.69 (4.16–10.76) (*p* < 0.001)
Model 2		1.00	1.94 (1.30–2.91) (*p* < 0.001)	6.69 (4.14–10.82) (*p* < 0.001)
Model 3		1.00	1.79 (1.19–2.69) (*p* = 0.005)	5.01 (3.02–8.34) (*p* < 0.001)

Six newly diagnosed chronic diseases were dependent variables, and sleep problems was the key independent variable. Model 1 only had variable sleep problems as the predictor; Model 2 further adjusted for age, gender and ethnicity; Model 3 further adjusted for education, NS-SEC, perceived current financial difficulty, perceived future financial difficulty, living with a partner, a dependent child under 16 in the household, COVID-19 symptoms, and any other baseline diagnosed chronic diseases.

## Data Availability

The survey data used for this study is available from UK Data Service https://ukdataservice.ac.uk/ upon application (accessed on 1 August 2022).

## Appendix A

**Table A1:** Crosstabulation between sleep problems, demo social characteristics, baseline chronic conditions and incidents of chronic disease.

	CVD	Hypertension	Diabetes	Obesity	Arthritis	An Emotional, Nervous or Psychiatric Problem
Sleep problems	*p* = 0.185	*p* < 0.001	*p* < 0.001	*p* < 0.001	*p* < 0.001	*p* < 0.001
Good sleeper	0.5	1.2	0.4	0.7	1.4	0.6
Moderate sleep problems	0.7	2.2	0.6	1.2	2.4	1.2
Severe sleep problems	1.2	3.2	1.5	2.1	3.8	4.1
Age	*p* < 0.001	*p* < 0.001	*p* = 0.004	*p* = 0.032	*p* < 0.001	*p* = 0.107
16–24	0.1	-	-	0.3	0.4	1.2
25–44	0.1	0.6	0.2	1.2	0.4	1.3
45–64	0.5	1.8	0.7	1.1	1.7	1.2
65–79	1.2	3.5	0.8	1.0	4.2	0.7
80+	4.4	2.9	1.2	-	6.7	0.3
Gender	*p* = 0.003	*p* = 0.941	*p* = 0.970	*p* = 0.008	*p* = 0.123	*p* = 0.156
Men	0.9	1.7	0.5	0.7	1.6	0.9
Women	0.4	1.7	0.6	1.2	2.2	1.2
Ethnicity	*p* = 0.066	*p* = 0.525	*p* = 0.781	*p* = 0.571	*p* = 0.133	*p* = 0.467
British white	0.7	1.7	0.6	1.0	2.0	1.1
Other white	0.6	1.7	0.4	1.1	2.7	0.6
BAME	0.1	1.7	0.6	1.2	1.3	0.8
Education	*p* = 0.707	*p* = 0.052	*p* = 0.006	*p* = 0.356	*p* = 0.001	*p* = 0.978
No qualification	0.6	2.1	0.6	0.9	2.9	1.0
GCSE	0.6	2.3	1.0	1.1	2.6	1.1
A level	0.7	1.8	0.4	1.3	2.2	1.0
Degree or above	0.7	1.4	0.4	0.9	1.6	1.1
NS-SEC	*p* = 0.014	*p* < 0.001	*p* = 0.004	*p* = 0.336	*p* < 0.001	*p* = 0.076
Professional	0.4	1.0	0.3	0.9	1.1	0.7
Intermediate	0.6	1.3	0.3	1.4	1.4	1.3
Routine	0.5	1.2	0.4	0.9	1.6	1.0
Not working	0.9	2.6	0.8	1.0	3.1	1.2
Perceived current financial difficulty	*p* = 0.037	*p* = 0.506	*p* < 0.001	*p* < 0.001	*p* = 0.158	*p* < 0.001
Living comfortably	1.0	1.6	0.4	0.6	1.9	0.6
Doing all right	0.5	1.6	0.4	0.8	1.8	0.9
Just getting by	0.6	2.0	1.1	1.8	2.5	1.9
Difficulty or very difficult	0.4	2.4	1.6	2.9	2.6	3.5
Perceived future financial difficulty	*p* = 0.491	*p* = 0.294	*p* = 0.203	*p* = 0.526	*p* = 0.030	*p* = 0.010
No change	0.7	1.8	0.5	1.0	2.0	1.0
Better off	0.4	1.0	0.5	1.0	0.9	1.1
Getting worse	0.8	1.4	1.0	0.9	2.4	2.1
Living with a partner	*p* = 0.569	*p* = 0.337	*p* = 0.411	*p* = 0.331	*p* = 0.049	*p* = 0.182
No	0.6	1.8	0.5	0.9	1.8	1.0
Yes	0.7	1.5	0.6	1.1	2.3	1.3
Dependent child under 16 in the household	*p* < 0.001	*p* < 0.001	*p* = 0.555	*p* = 0.980	*p* < 0.001	*p* = 0.467
No	0.8	2.0	0.6	1.0	2.4	1.0
Yes	0.1	0.8	0.5	1.0	0.6	1.2
COVID-19 symptoms	*p* = 0.798	*p* = 0.303	*p* = 0.721	*p* = 0.755	*p* = 0.871	*p* = 0.104
No	0.6	1.7	0.6	1.0	1.9	1.1
Yes	0.8	2.5	0.4	1.2	2.1	-
Baseline other chronic diseases	*p* < 0.001	*p* < 0.001	*p* = 0.002	*p* < 0.001	*p* < 0.001	*p* = 0.015
No	0.2	1.0	0.4	0.6	0.9	0.8
Yes	0.8	2.7	0.8	1.4	3.3	1.3
Total n who clears of baseline study disease	12,123	10,420	11,895	12,229	11,085	12,052
Study disease newly diagnosed n	78	176	66	121	216	129
% study disease incidence	0.7	1.7	0.6	1.0	1.9	1.1

Source: USoc: COVID-19 Study wave 4–9.

**Table A2:** Odds ratios (95% Confidence Intervals) for incidents of chronic disease according to the level of sleep problems over 15 months during the COVID-19 pandemic.

Incidents of Chronic Disease	Total *n*	Sleep Problems		
		Good Sleeper	Moderate Sleep Problems	Severe Sleep Problems
CVD (*n* = 77)	11,908			
Model 1		1.00	1.45 (0.90–2.35)	2.42 * (1.19–4.92)
Model 2		1.00	1.73 * (1.06–2.81)	3.13 ** (1.52–6.47)
Model 3		1.00	1.58 (0.97–2.58)	2.74 * (1.30–5.78)
Hypertension (*n* = 173)	10,226			
Model 1		1.00	1.92 *** (1.39–2.66)	2.92 *** (1.79–4.77)
Model 2		1.00	2.02 *** (1.45–2.81)	3.15 *** (1.91–5.20)
Model 3		1.00	1.91 *** (1.37–2.66)	2.61 *** (1.55–4.40)
Diabetes (*n* = 66)	11,682			
Model 1		1.00	1.54 (0.90–2.63)	3.87 ***(1.94–7.71)
Model 2		1.00	1.58 (0.92–2.71)	3.95 *** (1.97–7.95)
Model 3		1.00	1.41 (0.81–2.43)	2.72 ** (1.29–5.73)
Obesity (*n* = 118)	12,008			
Model 1		1.00	1.89** (1.27–2.80)	3.35 *** (1.90–5.89)
Model 2		1.00	1.79** (1.20–2.67)	3.08 *** (1.74–5.44)
Model 3		1.00	1.63* (1.09–2.43)	2.21 * (1.21–4.04)
Arthritis (*n* = 212)	10,881			
Model 1		1.00	1.73 *** (1.30–2.32)	2.84 *** (1.81–4.46)
Model 2		1.00	1.79 *** (1.33–2.41)	3.14 *** (1.97–4.99)
Model 3		1.00	1.64 *** (1.22–2.21)	2.47 *** (1.53–3.99)
An emotional, nervous or psychiatric problem (*n* = 125)	11,832			
Model 1		1.00	1.96 *** (1.30–2.94)	6.67 *** (4.12–10.79)
Model 2		1.00	1.93 *** (1.28–2.90)	6.69 *** (4.11–10.89)
Model 3		1.00	1.76 ** (1.17–2.67)	4.92 *** (2.93–8.25)

* *p* ≤ 0.05; ** *p ≤* 0.01; *** *p* ≤ 0.001. The sample was limited only to participants with 15 months of observations. Six newly diagnosed chronic diseases were dependent variables, and sleep problems was the key independent variable. Model 1 only had variable sleep problems as the predictor; Model 2 further adjusted for age, gender and ethnicity; Model 3 further adjusted for education, NS-SEC, perceived current financial difficulty, perceived future financial difficulty, living with a partner, a dependent child under 16 in the household, COVID-19 symptoms, and any other baseline diagnosed chronic diseases.

**Table A3:** Odds ratios (95% Confidence Intervals) for incidents of chronic disease according to the level of sleep problems over 15 months during the COVID-19 pandemic.

Incidents of Chronic Disease	Total *n*	Sleep Problems	
		Good Sleeper	Poor Sleep Quality
CVD (*n* = 77)	12,123		
Model 1		1.00	1.56 * (0.99–2.45)
Model 2		1.00	1.90 ** (1.20–3.00)
Model 3		1.00	1.72 * (1.08–2.74)
Hypertension (*n* = 173)	10,420		
Model 1		1.00	2.03 *** (1.50–2.76)
Model 2		1.00	2.15 *** (1.57–2.94)
Model 3		1.00	1.99 *** (1.45–2.73)
Diabetes (*n* = 66)	11,895		
Model 1		1.00	1.89 * (1.15–3.11)
Model 2		1.00	1.94 ** (1.18–3.21)
Model 3		1.00	1.63 (0.97–2.73)
Obesity (*n* = 118)	12,229		
Model 1		1.00	2.05 *** (1.42–2.96)
Model 2		1.00	1.93 *** (1.33–2.79)
Model 3		1.00	1.68 ** (1.15–2.46)
Arthritis (*n* = 212)	11,085		
Model 1		1.00	1.88 *** (1.43–2.47)
Model 2		1.00	1.97 *** (1.49–2.60)
Model 3		1.00	1.77 *** (1.33–2.35)
An emotional, nervous or psychiatric problem (*n* = 125)	12,052		
Model 1		1.00	2.64 *** (1.82–3.82)
Model 2		1.00	2.59 *** (1.78–3.75)
Model 3		1.00	2.24 *** (1.53–3.28)

* *p ≤* 0.05; ** *p ≤* 0.01; *** *p ≤* 0.001. Six newly diagnosed chronic diseases were dependent variables, and sleep problems was the key independent variable. Model 1 only had variable sleep problems as the predictor; Model 2 further adjusted for age, gender and ethnicity; Model 3 further adjusted for education, NS-SEC, perceived current financial difficulty, perceived future financial difficulty, living with a partner, a dependent child under 16 in the household, COVID-19 symptoms, and any other baseline diagnosed chronic diseases.
